# Sentinel Node Biopsy and Lumpectomy in a Patient with Machado–Joseph Disease

**DOI:** 10.1155/2019/2309598

**Published:** 2019-12-12

**Authors:** N. N. Aldawoodi, A. R. Escher Jr., A. Padalia, D. Padalia

**Affiliations:** ^1^Anesthesiology, H. Lee Moffitt Cancer Center and Research Institute, Tampa, FL, USA; ^2^Anesthesiology/Pain Medicine, H. Lee Moffitt Cancer Center and Research Institute, Tampa, FL, USA; ^3^Neurology, Barnes Jewish Hospital, St. Louis, MO, USA

## Abstract

Spinocerebellar ataxia 3 (SCA3), also known as Machado–Joseph disease (MJD) is an autosomal dominant, progressive neurodegenerative disorder. Patients present with cerebellar ataxia, dystonia, rigidity, and neuropathy that worsen with time. On a molecular level, it occurs due to a CAG trinucleotide repeat expansion in the *ATXN3* gene. Due to the risk of pulmonary aspiration, hypoventilation, autonomic and thermoregulatory dysfunction, vocal cord paralysis, progressive paraplegia, parkinsonian symptoms, and chronic pain, it has significant anesthesia implications. Rarely, case reports occur in the literature describing regional anesthetic management of patients with SCA3, but none that describe general anesthesia specifically with MJD. We therefore describe a case of a patient with SCA3 who successfully underwent general anesthesia and considerations for perioperative management of this patient population.

## 1. Introduction

Spinocerebellar ataxia 3 (SCA3), also known as Machado–Joseph disease (MJD) is an autosomal dominant, progressive neurodegenerative disorder. Patients present with cerebellar ataxia, dystonia, rigidity, and neuropathy that worsen with time. On a molecular level, it occurs due to a CAG trinucleotide repeat expansion in the *ATXN3* gene. Due to risk of pulmonary aspiration, hypoventilation, autonomic and thermoregulatory dysfunction, cases of vocal cord paralysis and progressive paraplegia, parkinsonian symptoms, and chronic pain, it has significant anesthesia implications [1]. There are three case reports in the literature describing regional anesthetic management of patients with SCA3, but none that describe general anesthesia specifically with MJD [2–4]. We therefore describe a case of a patient with MJD who successfully underwent general anesthesia and the considerations for perioperative management of this patient population.

## 2. Case Presentation

A 48-year-old female with breast cancer presented for right breast lumpectomy and axillary node dissection at an outpatient surgical center. She had the presumptive diagnosis of Machado–Joseph disease (MJD). She related an extensive family history of MJD in her maternal grandmother, maternal aunt, mother, two younger sisters, and her oldest daughter. Many of her family members passed away from complications of the disease. She recalled prior MRI and lumbar puncture but denied any genetic testing. Her other past medical history included hypertension, irritable bowel syndrome, and urinary incontinence. Her family history included several other relatives with this condition including her mother who passed away from issues related to the syndrome at 44 years old. Her preoperative laboratory studies and airway exam were unremarkable. Preoperative electrocardiogram (ECG) showed sinus rhythm with an incomplete left bundle branch block. Preoperative echocardiogram showed an ejection fraction of 60–65%, moderately dilated right ventricle with normal function, moderately dilated right atrium, moderate tricuspid valve regurgitation and right ventricular systolic pressure of 32 mmHg (millimeters of mercury) consistent with mild pulmonary hypertension. She was on tizanidine, a muscle relaxant for spasticity. A preoperative Neurology note indicated that the patient's MJD was “fairly advanced and she is currently not ambulatory” and while patient is not having “considerable choking… (her) speech is poor.” Another note from Neurology states she has “ataxia with severe dysarthria, limited extra-ocular movements, poor cough, some dystonic posturing and spasticity. She has tried dopaminergic drugs with no effect.”

On physical exam, her airway exam was unremarkable and she was noted to be a Mallampati 2 with good mouth opening of 5 centimeters. She had bilateral upper and lower extremity weakness and spasticity. Patient was wheelchair bound.

There was a literature search performed preoperatively, with no case reports found specifically detailing general anesthesia management in patients with MJD. Regional anesthesia was performed successfully in three case reports and therefore the patient was offered both a paravertebral and pectoralis block. The patient however declined these blocks despite the benefits being explained. Given that the surgeon would need to perform an extensive axillary node dissection, it was deemed that general anesthesia would be a more suitable anesthetic than local only infiltration per the surgeon. At that point, there was a discussion as to whether the case should be cancelled and rescheduled at an inpatient facility in case the patient was to experience perioperative complications. However, given scheduling issues and the time sensitive nature of this procedure in a patient with breast cancer, it was decided to proceed with a general anesthetic. Other case reports involving different types of spinocerebellar ataxia had detailed some considerations with general anesthesia which were followed.

No sedative premedication was given to avoid blunting airway responses. To avoid excessive post-operative opioids, the patient was given 1000 milligrams (mg) oral, acetaminophen pre-operatively. The patient was also given 20 mg of intravenous famotidine as an adjunct anti-nausea agent. The patient was brought back to the OR and standard ASA (American Society of Anesthesiologists) monitors were placed. The decision was made to place an LMA (Laryngeal Mask Airway) so neuromuscular blocking agents could be avoided. The patient was induced with propofol and a size 4 LMA Unique® was easily inserted. No opioid was given throughout the case as these patients are prone to hypoventilation and the surgeon generously infiltrated the surgical sites with local anesthetic. For maintenance of anesthesia, an intravenous propofol infusion was started to avoid volatile anesthetics and a SedLine® (Masimo) brain function monitor was placed on the patient's forehead to monitor depth of anesthesia. Given the pre-disposition of MJD patients to autonomic dysfunction and hypotension intraoperatively, a generous amount of intravenously fluids was given (about 1.5 liters for the case) along with frequent doses of phenylephrine and one dose of 0.2 mg of intravenous glycopyrrolate to avoid bradycardia and reduce secretions.

Dexamethasone and ondansetron were given intraoperatively for post-operative nausea and vomiting prophylaxis and to decrease the chance that an anti-dopaminergic anti-emetic would have to be administered post-operatively.

The patient was in the operating room for 1.5 hours. At the end of the case, the LMA was removed and patient was taken to the post-anesthesia care unit (PACU) on room air. She recovered uneventfully in the PACU and did not require any opioids. She was successfully discharged home after 1.5 hours.

## 3. Discussion

Spinocerebellar ataxias (SCAs) are a group of rare, hereditary, and progressive neurodegenerative disorders characterized by worsening cerebellar dysfunction—typically in the setting of many other neurological symptoms. Of the more than 30 subtypes of SCAs, Machado–Joseph Disease (MJD) or spinocerebellar ataxia type 3 (SCA3) is the most common form of SCA worldwide [5]. It is caused by an autosomal dominant CAG trinucleotide repeat expansion in the *ATXN3* gene. This results in the mutant ataxin 3 protein, which was previously a cytoplasmic protein, forming nuclear inclusions and causing cellular degeneration [6].

MJD is a multifocal neurodegenerative disorder that preferentially affects the cerebellum, pyramidal tracts, extrapyramidal tracts, motor neurons, and the oculomotor system. The common clinical presentation includes worsening cerebellar ataxia and an upper motor neuron syndrome including spasticity, hyperreflexia, and difficulty with fine motor skills [7]. However, this usually occurs with many other symptoms spanning from extrapyramidal signs to peripheral nerve dysfunction. In addition, onset of symptoms can occur anywhere from ages 10 to 70 with widely varying rates of progression [8]. Despite the phenotypic variability of MJD, there are many minor features that are typical for the disease including exophthalmos, progressive external ophthalmoplegia, facial and lingual fasciculations, and dystonia [7]. Like other SCAs, management of MJD is largely supportive and aimed at controlling symptoms of spasticity, Parkinsonism, dystonia, and myalgia; there are no effective treatments available for MJD.

Many disorders of the central nervous system can be considered relative contraindications to anesthesia due to the risk of exacerbating preexisting neurologic deficits. These risks are often difficult to stratify due to disease heterogeneity and the absence of robust literature surrounding the topic. With respect to MJD, there are only three case reports that describe regional anesthetic management and no case reports discussing general anesthesia [2]. Patients with MJD have numerous clinical features with important implications for anesthesia.

Dysphagia and vocal cord paralysis due to bulbar dysfunction predisposes MJD patients to aspiration, hypoxia, and delayed postoperative recovery of cough and gag reflexes. In fact, aspiration pneumonia is the most common cause of death in SCA patients [9]. This is exacerbated by peripheral denervation, which can cause respiratory muscle weakness [10]. As a result, regional anesthesia is preferred to general anesthesia due to the higher risks for aspiration and hypoxia in the latter. Moreover, there are numerous case reports supporting the safety of regional anesthesia for SCA including MJD [2, 3]. There is a theoretical risk for postoperative neuropathy when administering regional anesthesia, especially in patients with preexisting peripheral neuropathy, but this has not been reported in SCA [11]. In this instance, the patient refused regional anesthesia and local infiltration analgesia alone was inappropriate for the surgery. However, she did not have preexisting bulbar palsy, so general anesthesia was chosen despite the risks.

Similar respiratory concerns inform the avoidance of opiates to minimize risk of delayed hypoventilation. This emphasizes the selection of regional anesthesia including peripheral nerve blocks and neuraxial anesthesia when possible; this can be reinforced with multimodal nonopioid analgesia such as acetaminophen, celecoxib, and gabapentin. Our patient was given 1000 mg of oral acetaminophen and generous local infiltration analgesia and did not require postoperative opiates.

Dysautonomia can cause unpredictable and potentially life-threatening responses to general and neuraxial anesthesia including circulatory collapse after initiation of mechanical ventilation [12]. This supports stricter control over intravascular volume, blood pressure, and heart rate with fluids, vasopressors, and anticholinergics. An arterial line can be considered in longer cases for more accurate hemodynamic monitoring. Our patient was preemptively given a generous amount of intravenous fluids; she required frequent doses of phenylephrine and a single dose of glycopyrrolate to avoid hypotension and bradycardia.

Patients with MJD may be sensitive to medications that can alter neural physiology. Unfortunately, due to scant literature, guidelines must be extrapolated from case reports on patients with similar disorders. For instance, patients with Huntington's chorea—another inherited neurodegenerative disorder due to CAG repeats—can have exaggerated responses to barbiturates resulting in prolonged apnea and recovery time in patients. This may advise avoidance of premedication with barbiturates in patients with MJD [13]. Similarly, circulatory collapse after administration of succinylcholine has been reported in patients with diffuse lower motor neuron disease [14]. This may suggest avoiding depolarizing and nondepolarizing muscle relaxants in patients with MJD to minimize risk of protracted neuromuscular paralysis. We opted to use a laryngeal mask airway (LMA) in our patient to avoid the use of muscle relaxants. This may increase the risk of aspiration, but our patient did not have dysphagia, and there have been reports of using LMAs in SCA patients without airway complications [15].

Volatile anesthetics such as halothane and enflurane have been reported to alter cerebellar cGMP and subsequently affect motor activity in mice [16]. Volatile agents can also induce aberrant calcium release from the endoplasmic reticulum and cause neural cell damage; alterations in calcium homeostasis have been implicated in the pathogenesis of SCA [17]. Further, there is a case report describing refractory head tremor after volatile anesthesia in a patient with SCA6 [18]. Therefore, volatile anesthetics should be used carefully in patients with MJD. We used propofol for both induction and maintenance anesthesia in our patient to avoid using volatile anesthetics.

Anti-dopaminergic medications can potentially induce or exacerbate extrapyramidal symptoms including dyskinesia and dystonia in patients with MJD [19].This cautions against using medications such as prochlorperazine to treat postoperative nausea and vomiting. In our patient who had preexisting dystonia, we gave intraoperative dexamethasone and ondansetron as prophylaxis against postoperative nausea and vomiting.

MJD patients can often have emotional instability, cognitive dysfunction, and dysarthria, which can cause communication problems [20]. Additionally, patients with MJD often have myodystonia and involuntary movements, which can prevent proper positioning for procedures. Neuraxial and general anesthesia can alleviate spasticity in these patients, which allows for easier positioning.

## 4. Conclusion

In conclusion, we successfully administered general anesthesia to a patient with Machado–Joseph Disease. Certain patients with this disease process may potentially undergo general anesthesia safely.
